# Characterizing Brand Knowledge and Identification as Predictors of Consumer-Based Brand Equity: Mediating Role of Employee-Based Brand Equity

**DOI:** 10.3389/fpsyg.2022.858619

**Published:** 2022-04-27

**Authors:** Zijing He

**Affiliations:** School of Economics, Liaoning University, Shenyang, China

**Keywords:** brandequity, employee-based brand equity, consumer-based brand equity, brand identification, brand knowledge

## Abstract

Branding has been a key factor for the software houses, mainly customers’ expectations for a predicted product and real-time experience. The identity and knowledge of brands set a certain set of expectations in the mind of the consumers and the organization’s employees. This study mainly investigates the effects of brand identity and brand knowledge on the employee-based brand equity (EBBE) and consumer-based brand equity (CBBE). Further, it examined the mediating role of EBBE among these variables. To complete this empirical study, a quantitative survey was conducted using a 30-item survey method to collect data from 243 respondents from China’s software houses. The participants were selected based on purposive sampling. Results show that brand identity and brand knowledge are the main constituents of EBBE, which significantly predicts the CBBE. The study highlights the importance of employees in building overall brand equity. Training and brand promotion activities would help the organizations build a brand identity that positively contributes to the EBBE. Further, brand identity and brand knowledge are needed to improve the human capital, engagement of employees, and their emotional affiliations with the organizations, ultimately making the brand equity of employees stronger.

## Introduction

Branding has become an important aspect for software companies, particularly in terms of consumer expectations for an anticipated product and also real-time experience. The identity and knowledge of brands create a set of expectations in the customer’s mind and also workers of the firm. The competition among service brands has intensified. The problem of gaining and maintaining customers is made more difficult by the increasingly fragmented market caused by growing consumer knowledge and experience (Holiday et al., 2021). Brand love has begun to emerge as a fresh marketing trend that is gaining the attention of academia and also industry practitioners. Marketing professionals must maintain a favorable consumer-based brand equity (CBBE) (Kim J. et al., 2021). Each component of brand equity (physical quality, employee conduct, ideal internal consistency, brand identity, and lifestyle consistency) represents consumers’ views and imaginations of the brand, and the picture they build helps the consumer make purchase decisions (Avotra et al., 2021).

In marketing literature, CBBE is said to be a multidimensional concept (brand awareness and brand image). CBBE, or the overall instrumental value that customers give to a specific brand, is important in assessing long-term brand value (Kotsi et al., 2018). Because brand equity is built *via* organic interactions between the different components that constitute a brand. Understanding these relationships within the integrated frameworks of CBBE is critical for measuring branding success and effectively managing the brand. In recent years, the idea of CBBE has gained traction among academics. CBBE and its many characteristics are significant for businesses because of its impact on customer’s satisfaction, perceived benefits, and loyalty (Sürücü et al., 2019). Quality perception, brand identification, brand loyalty, and brand image are all ways to develop CBBE (Kotsi et al., 2018).

Customer-based brand equity is viewed as a multidimensional concept in brand management, despite debates about whether the ideas underpinning brand awareness development can be applied directly to brands like hotels, food, and beverage enterprises, where the service aspect is dominating (Rifi and Mostafa, 2021). Because the diverse elements of brand equity are exposed when brands are evaluated in the service industry, adaptations to service-based branding models are required to fulfill service supply’s particular features (i.e., non-physical). In their CBBE assessment in the service sector, successful brands are designed to suit physical customer desires and meet their symbolic needs (Wilson et al., 2021). Despite the fact that brand love has emerged as a key component in the consumer–brand relationship, few research studies have been conducted to date on the origins of the love connection between a customer and a brand, and also the potential behavioral implications (e.g., loyalty) (Maleki Minbashrazgah et al., 2021). Brand love, for example, may be influenced by the product or brand’s features (e.g., a hedonic product whose primary reward is amusement, joy, or pleasure), and it can also impact brand loyalty.

As a result, brand love may be seen as a catalyst for brand loyalty, which influences customers’ behavioral intentions and attitudes and their steadfast allegiance to the brand (Mody and Hanks, 2019). The previous research has shown a meaningful and positive association between brand love and brand loyalty (Kotsi et al., 2018). As a result, brand equity is critical to every institution’s development and survival. Many elements of its origins and consequences, on the other hand, remain unexplored and less understood. Despite its widespread in marketing, brand equity’s significance in managing service brands has been underestimated (Schmidt and Baumgarth, 2018). Its impacts on national (local) vs. international (foreign) brands are similarly understudied. There has been no earlier investigation of the impacts of brand equity, brand knowledge, and value perception on total brand equity, using brand loyalty as a mediating role (Glaveli, 2021). Brand equity has been developed in marketing as an “intangible asset and a critical value-driver of business success.” It may be observed from a variety of viewpoints, which includes those of financial markets, consumers, and workers (Zollo et al., 2020).

Several scholars have attempted to comprehend it through cognitive psychology. Kotsi proposed a third approach, CBBE, which is based on consumers’ brand knowledge, especially on the idea that the strength, favorability, and originality of brand awareness are stored in memory. This strategy is based on the distinct marketing benefits of brand knowledge derived through brand awareness (Kotsi et al., 2018). When customers’ recollections of one brand are more pleasant, stronger, or distinctive than those of another, CBBE arises. When consumers react more favorably to a company’s marketing mix, or any component of it than to a nameless or fictitious version of the same product, the brand shows positive CBBE. Resultantly, brand equity in this context is based on a position compared to a competing brand – whether genuine or simulated. Brand equity is intellectual capital created in the minds of workers *via* strong marketing and human resource activities. EBBE is the value that a brand adds to a company by influencing the attitudes and behaviors of its workers (Lee et al., 2019).

Consumer-based brand equity is becoming more of a concern, but the role of workers becomes more prominent (Christodoulides and Chernatony, 2010). Employees’ capacity to execute on client expectations is the foundation for building a strong brand and delivering perceived service excellence (King et al., 2013). As the focus of attention changes more and more to employees, experts argue that studying brand equity from the standpoint of employees, dubbed EBBE, is vital (Gounaris, 2006; Mo et al., 2021). Enhancing EBBE helps organizations recruit competent individuals, and workers’ skills and experience provide them a competitive edge. Professionals’ identification with enterprises, on the other hand, may add to client satisfaction since they connect directly with consumers or customers (Poulis and Wisker, 2016).

Employee-based brand equity, as defined by King, is “the unequal influence of brand knowledge on an employee’s responsiveness to internal brand management.” Moreover, how employees become related to brand values remains a key study subject. As a result, the notion of brand equity has grown in prominence as a prerequisite for effective internal brand management. In contrast, two widely accepted approaches on brand equity continue to dominate the literary works: customer-based and financial-based brand equity. That is why King and Grace came up with the idea for the third point of view. The authors advocated EBBE in their groundbreaking study, which emphasizes brand expertise as the cornerstone to inside brand building initiatives (King et al., 2013; Erkmen, 2018). Given the notion’s inception and the trend toward that third perspective for brand equity, most research to date has focused on the idea theoretically or conceptually (King and Grace, 2005, 2008, 2009, 2010; King et al., 2012). Based on this gap, this research focused on identifying the role of brand knowledge and brand identity on EBBE leading to CBBE.

Employee-based brand equity is a behavioral result of both corporate and also internal branding. Because the corporate brand is the primary concept of both employer branding and internal branding. Employer branding activities result in good employee behaviors aimed toward the brand image (Nogueira et al., 2020). Consumer-based brand identification refers to a person’s sense of belonging to a specific brand (Yoshida et al., 2021). Despite increased awareness, experts contend that there is always more to learn about the significance of customer-based brand identification and also its relationship to consumer’s behavior and branding (Li F. et al., 2021). Consumer identification affects individual consumers’ behavior, which includes purchasing decisions, brand preference, psychological sense of brand community and brand identification, the satisfaction of customers and a higher likelihood of repurchase, increased customer loyalty, and consumers’ decision to purchase premium (Niedermeier et al., 2021). Although previous research has provided valuable insights into the consumer identification process and associated dimensions (Ye et al., 2021), current research tried to fill in significant gaps in a way to explore the relationship between brand identification and CBBE.

## Theoretical Underpinning and Hypothesis Development

Brand knowledge is being used to develop overall brand recognition to the allocation of public consumption patterns, it is also utilized to put workers’ brand-related job behaviors in jeopardy. Similarly, EBBE refers to the employees’ identification with the brand. In the literature, there are two viewpoints to describe the employer-employee relationship: social exchange-based and brand identification-based relationships. Social exchange theory, which describes workplace relationships *via* the trade of physical resources, has evolved into a social exchange-oriented approach (Ashforth and Mael, 1989). Identification-based relationships, on the other hand, are founded here on social identity theory (SIT), which explains employee relationships as a match among personal and corporate identities (Erkmen, 2018). The SIT is the foundation of this research. The SIT was used in a variety of settings, which include the psychology of consumers, information dissemination, and the connection between sports franchises and their supporters (Dimofte et al., 2014; Mckinley et al., 2014; Ambrose and Schnitzlein, 2017).

Social identity theory is a core theory in cognitive science that has been used to explain group psychology, interacting, and social perspectives. It was proposed by Tajfel and Turner (2004). The component of one’s self-concept that stems out from social group or groups to which someone belongs, and also the significance and psychological value linked to affiliation to an organization, is referred to as social identity. It is the aspect of self-identity that is mostly generated from belonging to a group (Tajfel and Turner, 2004). People tend to associate and link themselves to different brands as a way of selecting self-identity and a feeling of belonging, according to the SIT. The personal self is founded on the importance and significance that an employee puts on brand identity, according to the theory. As a result, humans form a sense of social identity regarding the social characteristics of the brands to which they can relate (Chan, 2016).

### Brand Knowledge, Consumer-Based Brand Equity, and Employee-Based Brand Equity

Due to its significant function as an intellectual capital business asset during the last few decades, brand equity has been one of the primary focuses of interest for managers and marketing experts. There are several definitions of brand equity. According to one of the most frequently recognized definitions, brand equity is defined as the “added value conferred by the brand to the product” (Jeon and Yoo, 2021). Some researchers have come up with their definitions. A combination of brand assets and liabilities associated with a brand, its name, and symbol that increase or decrease the value supplied by a product or service to a company and its consumers are known as brand equity (Firmansyah et al., 2021). Keller defined brand equity as “the differential influence of brand knowledge and consumer reaction to the marketing of the brand,” which he defined as “the differential effect of brand knowledge on the consumer-based brand equity of the brand.” Brand equity is also described as “the increase in the perceived usefulness and desirability of a product conferred by a brand name” (Keller, 1993).

Employee-based brand equity and also CBBE are comparable in the sense that they are both values derived from the brand’s inherent character (Prados-Peña and Del Barrio-García, 2021). EBBE is estimated by the following influence that brand knowledge has on an employee’s reaction to his or her work settings and cultures and is characterized from the point of view of customers (Hanaysha and Al-Shaikh, 2021). A brand is any title, mark, symbol, or combination of these used to identify and differentiate a service or product from its rivals (Ali et al., 2021). From the perspective of consumers, brand knowledge is the awareness of the brand personality (Zhang et al., 2022). It comprises a guarantee from the company to its customers and typically depicts what the brand has endured (Cambra-Fierro et al., 2021). It also describes how people felt about the brand. Essentially, brand knowledge is a collection of concepts in the consumer’s mind about a certain brand (Kumar and Kaushal, 2021).

Furthermore, because it has actual and totally practical linkages with the psychology of customers, brand knowledge is the valuation of a product in the user’s memory (Li S. et al., 2021). In today’s business world, a company’s brand identity is viewed as an advantage since consumers are drawn to well-known brands that have a strong image in their minds (Islam et al., 2021). Finally, the brand identity symbolizes both basic principles and the business as a whole. Because the identity of a brand typically reveals its personality, it is incredibly significant for a firm (Jamshidi and Rousta, 2021). The brand identity also determines product sales since an appealing brand identity attracts buyers to purchase a product by making the choice process easier (Mehta and Tariq, 2020). Due to the high cost, increased competition, and low demand in today’s market, businesses are focusing their efforts on increasing the efficacy of their promotional expenditures (Krizanova et al., 2019). Because it is vital to have a thorough understanding of brand equity to improve market efficiency (Troiville et al., 2019).

Although vendors establish market knowledge about the brand in the minds of customers, it typically leads to CBBE, which solely validates Keller’s CBBE thesis (Pillay and Sibiya, 2021). Because of ephemeral market techniques, brand knowledge is heavily affected in the minds of end customers (Holiday et al., 2021). Brand knowledge is a complex tool since it enables consumers to recall information about a brand effectively and rapidly once they have learned about it (Chesbrough, 2020). Customers’ impressions are successfully impacted by brand knowledge since it pushes individuals to choose a brand based on prior knowledge (Yunpeng and Khan, 2021). Overall performance, elements, familiarity, sentiment, reflection, and brand repute are all influenced by brand knowledge (Kim J. et al., 2021). In the framework of brand promotions, the above-mentioned directions entailed establishing contacts with customers (Borges et al., 2021). The primary goal of such applications is to remind end customers about the most popular brands by leveraging their brand knowledge and wants during the purchasing process (Gielens et al., 2021).

Brand knowledge aids businesses in establishing a positive brand image in the eyes of customers (Törmälä and Saraniemi, 2018). It is also considered a competitive tool since when corporations send information about their brands to distributors, they can be confident that all of the information will be fully understood by the sellers, which gives them a competitive advantage (Górska-Warsewicz et al., 2021). It has a lot to do with all the brand’s relationships with customers. Keller’s CBBE model is completely compatible with the brand knowledge notion (Zarei et al., 2021). He emphasized that corporate image is a success factor since the organization’s strength is primarily dependent on how customers have encountered the brand and how fast they can recall it (Kapoor and Banerjee, 2021). According to this hypothesis, brand awareness improves consumer relationships in general (Chen et al., 2021).

Brand knowledge has such a strong impact on people’s thinking, customers may recollect their memories based on it (Zhou et al., 2021). Employee brand internalization guarantees that employees have a thorough understanding of brands and a strong commitment to them, reflecting cognitive and emotive pathways to EBBE (Maleki Minbashrazgah et al., 2021). Employees will encourage pro-brand activities after they acknowledge the organization’s aims and values (Binu Raj, 2021). The more a company’s ideas and principles of service are internalized, the more consistently and successfully employees will execute as a result (Sonmez Cakir and Adiguzel, 2022). Employees get better brand dedication, expertise, and engagement as a result of brand internalization, which leads to EBBE, and may then achieve or even surpass customers’ projected brand value (Barros-Arrieta and García-Cali, 2021). As a result, we proposed these hypotheses.

***H***_1_. *Brand knowledge has an association with CBBE*

***H***_2_. *Brand knowledge has an association with EBBE*

### Brand Identification, Consumer-Based Brand Equity, and Employee-Based Brand Equity

For years, brands have been critical in establishing long-term customer relationships and ensuring long-term economic success (Rovanto and Bask, 2021). Concerns of consumer–brand identification have become more and more crucial for brand management in this time of high consumer cynicism toward companies, and also the decline in the usefulness of conventional media in marketing brands and the present global economic crisis (Wilson et al., 2021). Consumer–brand identification refers to a person’s sense of belonging to a specific brand (Yoshida et al., 2021). Despite increased awareness, experts contend that there is always more to learn about the significance of customer-based brand identification and also its relationship to consumer behavior and branding (Li F. et al., 2021). Consumer identification affects individual consumers’ behaviors, which include purchasing decisions, brand preference, psychological sense of brand community and brand identification, the satisfaction of customers, and a higher likelihood of repurchase, increased customer loyalty, and consumers’ decision to purchase premium (Niedermeier et al., 2021). Although the previous research has provided valuable insights into the consumer identification process and associated dimensions, future research might fill in significant gaps in this field of study (Ye et al., 2021).

To begin with, much focus has been directed toward concepts related to consumers’ brand identification. There has been less effort to empirically document the determinants of consumers’ brand identification and to relate the concept of consumer identification with some other variables such as brand commitment and also positive word of mouth (WOM) (Rajaobelina et al., 2021). Second, the branding literature concentrates primarily on the notion of brand loyalty, rather than the concept of brand commitment, which is more commonly found in the relationship of marketing literature (Kumagai and Nagasawa, 2021). Consumers can associate with companies (and their brands) as relevant social categories (Hu et al., 2019). Consumers with stronger brand identification are more likely to engage in pro-brand activities such as supporting the company’s goals, trying to protect its public image, supporting its brands, and brand loyalty (Gill-Simmen et al., 2018). Therefore, we suggested these hypotheses.

***H***_3_. *Brand identification has an association with EBBE*

***H***_4_. *Brand identification has an association with CBBE*

### The Mediating Role of Employee-Based Brand Equity

The entire foundation for understanding CBBE is the idea of brand knowledge (Chatzipanagiotou et al., 2019). Similarly, workers’ brand knowledge is valued as a foundation for recognizing the value of internal branding initiatives in developing EBBE (Smith et al., 2021). Both ideas (CBBE and EBBE) are also focused on the brand’s intrinsic character (Brunetti et al., 2019). EBBE, on the other hand, describes the influence that brand awareness has on an employee’s reactions to the workplace (Boukis and Christodoulides, 2018). Employees may reduce job ambiguity, which is directly tied to their performance, by recognizing brand recognition (Call and Ployhart, 2020). As a result of the disparate and hazy understanding of what the organization’s brand implies, employees are likely to communicate confusing brand promises to consumers, thus destroying the organization’s brand equity (Glaveli, 2021).

The idea of EBBE has recently been a hot issue in the realm of brand equity literature and theories (Nogueira et al., 2020). The EEBE considers employee brand knowledge to become the cornerstone of generating CBBE, as it will enthuse them to serve consumers and fulfill the organization’s goals (Sürücü et al., 2019). Employees’ responses to the nature of the workplace are reflected in EBBE, which is highly dependent on the differential effect that brand knowledge has on them (Awan et al., 2017). Employees at the other end play a critical role in bridging and strengthening ties between consumers and firms (Peñalba-Aguirrezabalaga et al., 2021). However, due to a lack of focus on EBBE tools and metrics, there is no clear and widely acknowledged foundation for it (Olanipekun et al., 2021).

However, the CBBE concept has been described as a tool in a variety of external branding situations, which include advertising, vacation, sports, and also the fashion sector, whereas just a few studies have looked into the EBBE’s antecedents and repercussions (Kotsi et al., 2018). As a result, this study looks at EBBE *via* brand knowledge and brand identification (Siqueira et al., 2021). The previous literature on brand equity has mostly focused on CBBE, which is based on cognitive psychologists (Pina and Dias, 2021). Because when a brand has no significance or content of the product, it is ultimately useless for investors, manufacturers, or consumers. The CBBE concept is the prevailing situation and favored by overwhelming academicians and policymakers in consumer research (Kim E. J. et al., 2021). CBBE refers to brand equity that arises when a customer is familiar with the brand and has some favorable, powerful, and distinctive brand connections in their memory (Rifi and Mostafa, 2021).

Consumer-based brand equity refers to brand awareness that arises whenever a customer is aware of the brand and has some favorable, powerful, and distinctive brand connections in their psyche. Long-term revenues, consumers’ propensity to seek out new distribution channels for themselves, the power of enterprises to charge higher prices, and also the efficiency of marketing messages are all advantages of favorable CBBE (Jalalzadeh et al., 2021). CBBE is described in the literature as a decision-making tool that provides managers with a helpful diagnostic of consumers’ perceptions of the company (Fernández-Ruano et al., 2022). CBBE is best described as a construct resulting from brand-related connections, with the influence of those linkages concentrated (Cruz-Milán, 2021). We need a deeper knowledge of the composition of brand equity in different cultural settings and different product categories to give advice to management on how to handle their brand equity or investigate the network of its component elements. As a result, we purposed these hypotheses to explain the mediating relationship of brand knowledge, brand identification, and CBBE through EBBE.

***H***_5_. *EBBE mediates the relationship of brand knowledge and CBBE*

***H***_6_. *EBBE mediates the relationship of brand identification and CBBE*

**FIGURE 1:**
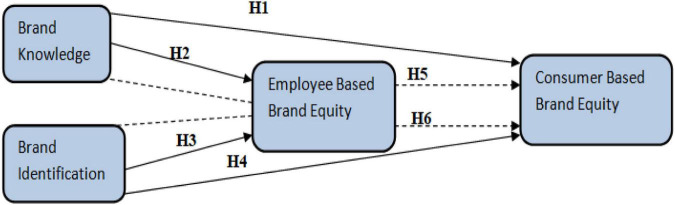
Conceptual model.

Based on the above hypothesis and literature following framework (see Figure 1) has been developed.

## Methodology

This study has applied the quantitative method with a deductive approach to analyze the data. The research philosophy followed here is post-positivism (Avotra et al., 2021), because the relationships of hypotheses have been checked as cause and effect of identified independent variables on other variables. The data are collected through questionnaire surveys from the employees of software houses in China. The population used in this study are the employees of software houses in China. The sample has been selected through the convenience sampling method (Yingfei et al., 2021). A major reason for using convenience sampling is the post-covid situation where people are maintaining social distancing and avoiding direct physical interaction with other people (Serafini et al., 2020). This is a non-probability sampling method in which the sample is selected based on the convenience of the researcher (Xialong et al., 2021). Prior consent had been taken from the potential participants for their availability by contacting the project managers of different software houses located in the mainland of China.

A total of 400 questionnaires had been dropped at the software houses and were explained about the questionnaire to the manager for any misunderstanding or ambiguity. The questionnaire was in the English language and it was ensured that the potential participants properly understand the questionnaire. The anonymity of the respondents had been ensured and the respondents were encouraged to respond independently without being under influence of the organizational authorities. The organizations were visited after 2 weeks to collect the questionnaires. Only 131 questionnaires were filled by them, whereas the rest of 112 questionnaires were collected a week later. The total questionnaires obtained after 3 weeks were 258, whereas the usable questionnaires were 243 making a response rate of 60.75%. The unit of analysis of the study is the employees of the software houses in China. The ethical consideration has been considered while collecting the data by not forcing the participants to return the questionnaires right then.

The ease and comfort of the respondents had been given preference. The data obtained from the surveys had been analyzed using the partial least square structural equation (PLS-SEM) modeling. Through SEM analysis, the data had been analyzed in two steps. The first step checks the validity and reliability of the data obtained, whereas in the second step, the hypotheses developed in the study have been checked to identify whether the data support or does not support them. PLS-SEM analysis is robust with model estimation, especially for theory development. This study has used this statistical tool as it has proposed certain hypothesis forming the basis for the theory.

### Common Method Bias

The common method variance, in the study, has been checked with Harman’s one-factor test using SPSS 26. Initially, the biases in the responses have been checked with Harman’s one-factor test since the responses obtained through convenience sampling can create biasness. This can be considered as under control if the survey method has been ensured for the understanding of the respondents Sharma et al. (2009) which has been incorporated in this study. The one-factor variance obtained from the factor reduction method has been reported as less than 50% Abbas and Sağsan (2019); hence, no statistical indication for the biases in the responses is found. The variance obtained for one factor for this study was 46%. The results can be seen in Appendix Table A1.

### Statistical Tool

This study has employed the PLS-SEM through Smart-PLS and SPSS software for analyzing the data. This is a statistical tool that analyzes the complex causal effects in the form of path models. It provides less contradiction among the results than regression analysis (Ramli et al., 2018). Quantitative statistical tools have been used to analyze in two stages: preliminary screening and hypothesis testing. First of all, the data were ensured for rationality and the absence of bias through employing Harman’s one-factor test. This test was conducted using the factor reduction method for common method bias in SPSS. In the first stage, the measurement model was assessed for the reliability and validity of data. In the second stage of the structural model, outcomes are obtained that are based on the covariance-based structural equation modeling. For hypotheses testing t-statistics, *p*-Values, R-square, and f-square statistics have been employed.

### Measurement

In this study, the questionnaires were used as the survey instrument. It consisted of 30 items in total relating to each variable of the study. There were four variables, which include two independent variables, one mediating variable, and one dependent variable. The acceptable value for Cronbach’s alpha is reported as 0.7 in the literature (Shah Alam and Mohamed Sayuti, 2011). All these scales were compiled on a five-point Likert scale that ranged from 1 to 5. In the response category, 1 exhibited strongly disagree, 2 exhibited agree, 3 showed a neutral response, 4 exhibited agree, and 5 exhibited strongly disagree.

#### Brand Knowledge

The scale for the first independent variable of brand knowledge consisted of five items, and it has been adapted from the study of Yoo and Donthu (2001). The sample items included “I can recognize my organization among other competing brands.” The Cronbach’s alpha reliability obtained is 0.922, which is according to the acceptable range of alpha.

#### Brand Identity

The other independent variable (brand identity) consisted of eight items that have been adapted from the study of Liu et al. (2020). The sample items included “Our office layout, logo, and clothing represent our brand values.” The Cronbach’s alpha reliability obtained for brand identity is 0.923.

#### Employee-Based Brand Equity

The mediating variable of EBBE consists of thirteen items that have been adapted from King and Grace (2010). The sample items included “I am proud to be a part of the organization I work for.” The Cronbach’s alpha reliability for variable EBBE is 0.947.

#### Consumer-Based Brand Equity

The CBBE, the dependent variable, consists of four items that were also adapted from the study of Liu et al. (2020). The sample items included “Our brand is better known than our most important competitors.” The Cronbach’s alpha reliability for the variable EBBE is 0.918.

### Demographic Details

The demographic information of the respondents had been collected from the last part of the questionnaire that consisted of four categories, namely, gender, age, education, and nature of the job. The question on gender was categorized into two main streams, men and women. Among the respondents, 53.9% were men and 46.1% were women. Of the age categories, the highest numbers of respondents were between the ages of 21–25 years, followed by the category of 26–30 years, and the least number of respondents fell under the category of 31 and above. Considering the education factor of the respondents, around 15% of the respondents had Ph.D. or other diplomas or certifications, whereas the rest of the respondents were bachelor’s and master’s degrees holders, that is, 42% and 43%, respectively. Regarding the nature of the job of the respondents, 37.5% of respondents were software developers, 48.55% were web developers whereas the rest of them were from the human resource department. The results of the demographic profile can be seen in Table 1.

**TABLE 1:** Demographic analysis.

Demographics	Frequency	Percentage
**Gender**		
Male	131	53.90%
Female	112	46.09%
**Age**		
21 to 25	110	45.26%
26 to 30	72	29.62%
31 and above	61	25.10%
**Education**		
Bachelors	102	41.97%
Masters	105	43.21%
Ph.D. and others	36	14.81%
**Nature of Job**		
Software Development	91	37.44%
Web Development	118	48.55%
Human Resource	34	14%

*N = 243.*

## Data Analysis and Results

### Model Measurement

**FIGURE 2:**
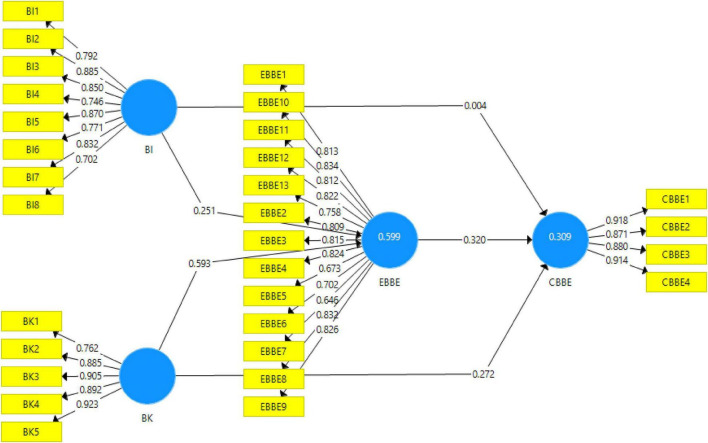
The output of the measurement model.

In the first stage, measurement model outcomes are used for checking the validities, reliabilities, average variance extracted (AVE), and factor loads for the initial screening to check whether the data are fit for hypothesis testing or not. For reliability of the data, Cronbach’s alpha and composite reliabilities are used whereas for validities, heterotrait–monotrait (HTMT) ratios and Fornell and Larcker criterion have been used. The algorithm obtained for the measurement model from the Smart-PLS software has been given in Figure 2.

The results obtained for factor loadings and AVE have been reported in Table 2 along with reliabilities. According to the study of Jogezai et al. (2021), the minimum values for the factor loadings for the items to be included in the scale have been reported as 0.65 whereas for AVE is 0.5. All the items of the study showed factor loadings well above the cutoff value except for the item EBBE7. The item EBBE7 showed the factor loading for less than the mentioned threshold; therefore, it was excluded from analysis for hypothesis testing. The factor loading of the items included in the study ranged from 0.673 to 0.918. On the other hand, all the values obtained for AVE were well above 0.5, thus showing the validity of the data. The values of AVE in this study ranged from 0.616 to 0.842 and were acceptable. Furthermore, the suitable range for the reliability has been mentioned as 0.7 (Jogezai et al., 2021); the results of the study have shown all the values of Cronbach’s alpha reliability and composite reliability above 0.7.

**TABLE 2:** Measurement model.

Variables	Factor Loadings		Cronbach’s alpha	CR	AVE
Brand Knowledge	BK1	0.762	**0.922**	**0.942**	**0.766**
	BK2	0.885			
	BK3	0.905			
	BK4	0.892			
	BK5	0.923			
Brand Identification	BI1	0.792	**0.923**	**0.937**	**0.653**
	BI2	0.885			
	BI3	0.850			
	BI4	0.745			
	BI5	0.870			
	BI6	0.771			
	BI7	0.832			
	BI8	0.702			
Consumer-based Brand Equity	CBBE1	0.918	**0.918**	**0.942**	**0.803**
	CBBE2	0.871			
	CBBE3	0.880			
	CBBE4	0.914			
Employee-based Brand Equity	EBBE1	0.813	**0.947**	**0.954**	**0.616**
	EBBE10	0.834			
	EBBE11	0.812			
	EBBE12	0.822			
	EBBE13	0.758			
	EBBE2	0.809			
	EBBE3	0.815			
	EBBE4	0.824			
	EBBE5	0.673			
	EBBE6	0.702			
	EBBE7	0.646			
	EBBE8	0.832			
	EBBE9	0.826			

*BI, brand identification; BK, brand knowledge; EBBE, employee-based brand equity; CBBE, consumer-based brand equity. Bold values shows the variable relationship.*

The other measures for validity used in this study are the HTMT ratio and Fornell and Larcker criterion (Fornell and Larcker, 1981). The acceptability for the validity of the data is considered if the values in HTMT ratios are below 0.85 (Franke and Sarstedt, 2019). In addition, for Fornell and Larcker criterion, each column should have the highest statistics at the top (Henseler et al., 2015). In this study, the values of the HTMT ratio are all significant and are all below 0.85; the highest value in the grid is 0.793 between the variables of brand knowledge and EBBE. These results have been mentioned in Table 3. Similarly, regarding the Fornell and Larcker criterion, all the highest values in each column are at the top that shows the discriminant validity of the data. The results for Fornell and Larcker criterion have been presented in Table 4.

**TABLE 3:** Discriminant validity (HTMT ratio).

	BI	BK	CBBE	EBBE
BI				
BK	0.665			
CBBE	0.398	0.552		
EBBE	0.651	0.793	0.561	

*BI, brand identification; BK, brand knowledge; EBBE, employee-based brand equity; CBBE, consumer-based brand equity.*

**TABLE 4:** Discriminant validity (Fornell and Larcker criteria).

	BI	BK	CBBE	EBBE
BI	**0.808**			
BK	0.618	**0.875**		
CBBE	0.370	0.514	**0.896**	
EBBE	0.618	0.748	0.526	**0.785**

*BI, brand identification; BK, brand knowledge; EBBE, employee-based brand equity; CBBE, consumer-based brand equity. Bold values shows the relationship.*

R-square, also known as the coefficient of determination, indicates the fitness of the proposed model by explaining the variance explained by each endogenous variable in the context of regression analysis. The R-square values obtained for the dependent variables in this study are all good. The variance of the variable EBBE has been 59.9% explained by the independent variables brand identity and brand knowledge. Similarly, the CBBE has been 31% explained by EBBE. Q-square shows the predictive relevance if the model shows predictive relevancy or not (the endogenous variables indicate any predictive relevance). A value of Q-square above zero shows that variables and the model possess predictive relevance. The endogenous variables in this study shows good predictive relevance with CBBE that shows 0.436 and EBBE that shows 0.373 values, thus meeting the criteria for predictive relevance of the model. The outer variance inflation factor values have been mentioned in Table 5. The threshold for VIF has been mentioned to be less than 5 (Craney and Surles, 2007). All the values obtained in this study are below this threshold, hence indicating the absence of multicollinearity. The results of VIF have been presented in Table 6.

**TABLE 5:** Outer VIF.

Variables	Item	Outer VIF
Brand Knowledge	BK1	1.701
	BK2	3.303
	BK3	3.603
	BK4	3.938
	BK5	4.759
Brand Identification	BI1	3.006
	BI2	4.028
	BI3	3.723
	BI4	2.175
	BI5	3.695
	BI6	2.454
	BI7	3.183
	BI8	1.690
Consumer-based Brand Equity	CBBE1	3.390
	CBBE2	2.571
	CBBE3	2.731
	CBBE4	3.441
Employee-based Brand Equity	EBBE1	3.945
	EBBE10	3.593
	EBBE11	3.246
	EBBE12	3.528
	EBBE13	3.011
	EBBE2	4.308
	EBBE3	4.562
	EBBE4	3.788
	EBBE5	1.890
	EBBE6	2.523
	EBBE7	2.307
	EBBE8	4.881
	EBBE9	4.737

*BI, brand identification; BK, brand knowledge; EBBE, employee-based brand equity; CBBE, consumer-based brand equity.*

**TABLE 6:** Direct effects.

Paths	H	β	T-Statistic	q-Square	f-square	*p*-Value	Results
BK→CBBE	H_1_	0.272	2.928	0.436	0.124	0.004	***Accepted***
BK→EBBE	H_2_	0.593	9.864	0.373	0.542	0.000	***Accepted***
BI→EBBE	H_3_	0.251	4.125		0.173	0.000	***Accepted***
BI→CBBE	H_4_	0.004	0.067		0.000	0.947	*Rejected*

*H, hypothesis; O, original sample; M, sample mean; SD, standard deviation; BI, brand identification; BK, brand knowledge; EBBE, employee-based brand equity; CBBE, consumer-based brand equity. Bold values shows the relationship.*

### Structural Model Estimation

The analysis obtained from Smart-PLS software gives the output for the results of the structural model. Based on the results obtained from the structural model, the decision for acceptance or rejection is taken considering the t-statistics and *p*-values. The results obtained from the structural model have been reported in Table 6 for the direct effects. These results show that the first and second hypotheses regarding the impact of brand knowledge on CBBE (β = 0.272, t-statistics = 2.92) and EBBE (β = 0.593, t-statistics = 9.86) have been accepted at *p* < 0.05. The third hypothesis showed a significant impact of brand identity on the EBBE (β = 0.251, t-statistic = 4.125, *p* < 0.00), thus accepting H_3_ whereas fourth direct effect (H_4_) has been rejected, which indicates no significant impact of brand identity on the CBBE.

Table 7 shows the results for the indirect effects of the study. Mediation of the study has been signified with the variance accounted for (VAF) values. According to the study of Pradhan et al. (2020) if the value of VAF obtained less than 0.20 shows no mediation, then values between 0.20 and 0.80 show partial mediation whereas above 0.80 shows full mediation. In this study, the first indirect effect of the study (H_5_) is regarding the mediation of EBBE between brand knowledge and CBBE, which has been accepted at *p* < 0.05 (β = 0.190, t-statistics = 3.05) and shows partial mediation (VAF = 41%) as the obtained values lie between 0.20 and 0.80. Similarly, the second mediation of the study has also been accepted, which shows a significant mediating effect of EBBE between the brand identification and CBBE (β = 0.081, t-statistics = 2.45, *p* < 0.05), which shows full mediation, VAF > 0.80.

**TABLE 7:** Indirect effects.

Paths	H	β	T-Statistic	Indirect Effect	Total Effect	VAF	*p*-Value	Results
BK→EBBE →CBBE	H_5_	0.190	3.057	0.189	0.461	41.0%	0.002	***Partial Mediation***
BI→EBBE →CBBE	H_6_	0.081	2.457	0.080	0.084	95.2%	0.014	***Full Mediation***

*H, hypothesis; O, original sample; M, sample mean; SD, standard deviation; BI, brand identification; BK, brand knowledge; EBBE, employee-based brand equity; CBBE, consumer-based brand equity; VAF, variance accounted for. Bold values shows the relationship.*

## Discussion

This research has been conducted to evaluate the directional relationship of brand knowledge and brand identification with CBBE. Moreover, the indirect role of EBBE was also evaluated among brand knowledge, brand identification, and CBBE. It is understood that EBBE assists workers in developing a link between their organization’s perceived financial cost and advantages and how it will influence them in the future. Whether there is an EBBE among the employees, the organization can profit from it in a variety of ways. This study demonstrated that software houses may effectively establish their CBBE with the support of a strong brand knowledge among the industry and stakeholders by taking use of the consumer-provided facilities. Similarly, good brand knowledge among software engineers inside software businesses may assist in enhancing their EBBE. Internally (EBBE) and externally (CBBE) brand equity is equally vital for the success of any brand in the IT business, which requires a combination of experience, abilities, and implementation.

This study empirically tested the mediating effect of EBBE on CBBE based on two critical variables to understand how brand identification and brand knowledge could contribute toward CBBE. These findings indicated that the first two hypotheses were accepted, which suggests that brand knowledge had a substantial influence on CBBE along with having a significant impact on EBBE. Basically, employees play a critical role in establishing total brand equity. Employees’ brand knowledge aids in their comprehension of the brand, which in turn aids in the reinforcement of their brand equity. Similar kinds of results have been reported in the past which suggests that knowledge or information about the brands among employees and consumers has a positive contribution toward creating equity of the brands whether it be employee-based or consumer-based. This is due to the fact that brand knowledge has such a strong impact on people’s thinking, customers may recollect their memories based on it (Zhou et al., 2021).

Employee brand internalization guarantees that employees have a thorough understanding of brands and a strong commitment to them, which reflects cognitive and emotive pathways to EBBE (Maleki Minbashrazgah et al., 2021). Employees will encourage pro-brand activities after they acknowledge the organization’s aims and values (Binu Raj, 2021). The more a company’s ideas and principles of service are internalized, the more consistently and successfully employees will execute as a result (Sonmez Cakir and Adiguzel, 2022). The next two hypotheses that indicate the directional relationship of brand identification with CBBEE and EBBE proved to be a strong determinant of brand equity. The results indicated that the determiner of brand equity, i.e., brand identification had a significant contribution in developing EBBE while it could develop a directional relationship with CBBE. Consumer–brand identification refers to a person’s sense of belonging to a specific brand (Yoshida et al., 2021).

The possible reason behind developing such internal organizational equity among employees is the fact that if employees are well equipped with the information about brand and brand imagery or the conception of the brand, then it develops a stronger sense of ownership among themselves. This could lead to a better EBBE, whereas the identification of brands by the consumers did not have a significant impact on CBBE due to the possible reasoning that employees are more concerned with their brands compared to the loyal consumers with brands which develops into a non-significant type of interaction between brand identification and CBBE. Some contrasting results are obtained in the previous studies, which indicate that brand identification could have a positive impact on developing CBBE (Yoshida et al., 2021). The indirect effects of EBBE proved to be a helping hand among brand knowledge, brand identification, and CBBE.

As reported in many studies that brand knowledge has an association with EBBE as an internal protocol, it was evident that EBBE could further strengthen the relationship of brand knowledge with CBBE. It is only possible due to the upholding ability of knowledge about the brand by the employees which helps in strengthening the brand equity at the consumer level. Even though the term brand knowledge refers to customers, the idea is also applicable to employees of software houses because brand awareness is the cornerstone for building brand equity. Likewise, employees who are familiar with the brand are more likely to grasp their responsibilities and execute the brand promise (Mangold and Miles, 2007; Erkmen, 2018). The direct effects between brand identification and CBBE were not significant and needed help of a mediator which could develop strong brand equity at the consumer level, so EBBE provided a stronger mediating link between brand identification and CBBE. This is also due to the strength of internal control, which leads to developing CBBE at the consumer level.

### Managerial Implications

Employee-based brand equity helps the employees to develop a relationship between the perceived costs and benefits of their organization and how it will affect them in the future. If there exists an EBBE among the employees, the organizations can reap certain benefits from it. (i) First of all, this study provides evidence that software houses can successfully build their CBBE with the help of a strong brand knowledge among the industry and stakeholders by taking advantage of the facilities provided to the consumers. (ii) Similarly, within the software houses, adequate brand knowledge among the software engineers can help strengthen their EBBE providing attractive compensations for their services.

(iii) Moreover, the management of other organizations can use this empirical evidence given by this study that the availability of brand knowledge among the employees and consumers can add value in the intensification of EBBE and CBBE, respectively. (iv) Turnover has been a challenge for the software industry which can affect the EBBE and consequently the CBBE. To cope with this challenge, continuous investment in the training, brand identity, and brand knowledge is needed so to improve the human capital, engagement of employees, and their emotional affiliations, which would ultimately make the brand equity of employees stronger. Training and brand promotion activities would help the organizations in building a brand identity that positively contributes to the EBBE.

### Theoretical Contribution

This study contributes in theory as it has found that EBBE’s role as a mediator between the brand knowledge, brand identification, and CBBE conveys the value of the brand from the organization to the final consumers, which leads to CBBE. Baumgarth and Schmidt (2010) highlights have been proven empirically in this study. It also highlights the important role that employees play in building overall brand equity for their organizations. The study also contributes theoretically by finding the employees’ brand knowledge and understanding of the brands they represent working in the organization help in their understanding of the brand that consequently helps in reinforcing their organizational brand equity.

### Limitations and Future Directions

Despite contributions to the literature, there are few limitations in the study that open new avenues for future research. First of all, the sample size is relatively smaller which is 243, and the results can be checked for generalizability with bigger sample size. Second, the software houses’ employees have been taken as the sample in this study; it is highly encouraged to conduct this study in other fields of professions, which considers different cultures for comparison and contrast to this study. Finally, some possible variables are expected to moderate the relationship between EBBE and CBBE (e.g., trust, customer’s satisfaction, customer care, brand image, etc.); hence, future studies should consider these variables as the moderators and check their effects on this kind of relationships.

## Conclusion

In the IT industry which needs a blend of expertise, skills, and execution, both internal (EBBE) and external (CBBE) brand equities are equally important for the success of any organization. This study has investigated the two key variables, that is, brand identity and brand knowledge to understand the mechanism of how these independent variables influence the EBBE and CBBE. The results of the study have indicated that brand identity positively and significantly affects the EBBE; however, it could not find any effect on the CBBE. Furthermore, brand knowledge has been found as a significant predictor of EBBE and CBBE. In addition, EBBE has been found as a significant mediator among the independent variables of brand identity and CBBE. Among the second relationship of the study between brand knowledge and CBBE, the EBBE has been found to partially mediate the relationship.

## Data Availability Statement

The original contributions presented in the study are included in the article/supplementary material, further inquiries can be directed to the corresponding author/s.

## Ethics Statement

The studies that involve human participants were reviewed and approved by the Liaoning University, China. The patients or participants provided their written informed consent to participate in this study. The study was conducted in accordance with the Declaration of Helsinki.

## Author Contributions

ZH conceived, designed the concept, collected the data, wrote the manuscript, and read and agreed to the published version of the manuscript.

## Conflict of Interest

The author declares that the research was conducted in the absence of any commercial or financial relationships that could be construed as a potential conflict of interest.

## Publisher’s Note

All claims expressed in this article are solely those of the authors and do not necessarily represent those of their affiliated organizations, or those of the publisher, the editors and the reviewers. Any product that may be evaluated in this article, or claim that may be made by its manufacturer, is not guaranteed or endorsed by the publisher.

## Appendix

**TABLE A1:** Total variance explained.

Total Variance Explained						
Factor	Initial Eigen values			Extraction Sums of Squared Loadings		
	Total	% of Variance	Cumulative%	Total	% of Variance	Cumulative%
1	14.428	48.094	48.094	13.916	46.385	46.385
2	2.814	9.380	57.474			
3	2.105	7.017	64.491			
4	1.644	5.480	69.971			
5	0.974	3.248	73.219			
6	0.939	3.128	76.348			
7	0.730	2.432	78.780			
8	0.638	2.125	80.905			
9	0.596	1.985	82.891			
10	0.573	1.911	84.802			
11	0.463	1.544	86.346			
12	0.428	1.426	87.772			
13	0.392	1.308	89.080			
14	0.358	1.192	90.273			
15	0.336	1.118	91.391			
16	0.306	1.022	92.413			
17	0.270	0.900	93.313			
18	0.255	0.850	94.162			
19	0.233	0.777	94.939			
20	0.215	0.716	95.656			
21	0.206	0.688	96.344			
22	0.186	0.621	96.965			
23	0.185	0.616	97.580			
24	0.155	0.517	98.097			
25	0.135	0.449	98.546			
26	0.119	0.396	98.942			
27	0.108	0.361	99.303			
28	0.088	0.292	99.595			
29	0.062	0.207	99.803			
30	0.059	0.197	100.000			

*N = 243.*

## References

[B1] Abbas J. Sağsan M. (2019). Impact of knowledge management practices on green innovation and corporate sustainable development: a structural analysis. *J. Clean. Prod.* 229 611–620. 10.1016/j.jclepro.2019.05.024

[B2] Ali G. Abbas S. Qamer F. M. Irteza S. (2021). Environmental spatial heterogeneity of the impacts of COVID-19 on the Top-20 metropolitan cities of Asia-Pacific. *Sci. Rep.* 11:20339. 10.1038/s41598-021-99546-9 34645879 PMC8514535

[B3] Ambrose S. C. Schnitzlein N. (2017). What makes for the best rivalries in individual sports and how can marketers capitalize on them. *Sport Mark. Q.* 26 223–234.

[B4] Ashforth B. E. Mael F. (1989). Social identity theory and the organization. *Acad. Manag. Rev.* 14 20–39. 10.5465/amr.1989.4278999

[B5] Avotra A. A. R. N. Chengang Y. Sandra Marcelline T. R. Asad A. Yingfei Y. (2021). Examining the impact of E-Government on corporate social responsibility performance: the mediating effect of mandatory corporate social responsibility policy, corruption, and information and communication technologies development during the COVID era. *Front. Psychol.* 12:737100. 10.3389/fpsyg.2021.737100 34712183 PMC8545817

[B6] Awan T. M. Li X. Haizhong W. (2017). Factors affecting employee-based brand equity: evidence from China. *Int. J. Manag. Stud.* 25 1–20. 10.32890/ijms.25.1.2018.10482

[B7] Barros-Arrieta D. García-Cali E. (2021). Internal branding: conceptualization from a literature review and opportunities for future research. *J. Brand Manag.* 28 133–151. 10.1057/s41262-020-00219-1

[B8] Baumgarth C. Schmidt M. (2010). How strong is the business-to-business brand in the workforce? An empirically-tested model of ‘internal brand equity’ in a business-to-business setting. *Ind. Mark. Manag.* 39 1250–1260. 10.1016/j.indmarman.2010.02.022

[B9] Binu Raj A. (2021). Internal branding, employees’ brand commitment and moderation role of transformational leadership: an empirical study in Indian telecommunication context. *Asia-Pacific J. Bus. Adm [Online ahead of print]* 10.1108/APJBA-04-2021-0175

[B10] Borges A. F. S. Laurindo F. J. B. Spínola M. M. Gonçalves R. F. Mattos C. A. (2021). The strategic use of artificial intelligence in the digital era: systematic literature review and future research directions. *Int. J. Inf. Manage.* 57:102225. 10.1016/j.ijinfomgt.2020.102225

[B11] Boukis A. Christodoulides G. (2018). Investigating key antecedents and outcomes of employee-based brand equity. *Eur. Manag. Rev.* 17 41–55. 10.1111/emre.12327

[B12] Brunetti F. Confente I. Kaufmann H. R. (2019). The human dimension of a brand influences brand equity: an empirical examination in the context of a luxury and a convenience brand. *J. Brand Manag.* 26 634–645. 10.1057/s41262-019-00162-w

[B13] Call M. L. Ployhart R. E. (2020). A theory of firm value capture from employee job performance: a multidisciplinary perspective. *Acad. Manag. Rev.* 46 572–590. 10.5465/amr.2018.0103

[B14] Cambra-Fierro J. J. Fuentes-Blasco M. Huerta-Álvarez R. Olavarría A. (2021). Customer-based brand equity and customer engagement in experiential services: insights from an emerging economy. *Serv. Bus.* 15 467–491. 10.1007/s11628-021-00448-7

[B15] Chan M. (2016). Social network sites and political engagement: exploring the impact of facebook connections and uses on political protest and participation. *Mass Commun. Soc.* 19 430–451. 10.1080/15205436.2016.1161803

[B16] Chatzipanagiotou K. Christodoulides G. Veloutsou C. (2019). Managing the consumer-based brand equity process: a cross-cultural perspective. *Int. Bus. Rev.* 28 328–343. 10.1016/j.ibusrev.2018.10.005

[B17] Chen X. You E. S. Lee T. J. Li X. (2021). The influence of historical nostalgia on a heritage destination’s brand authenticity, brand attachment, and brand equity. *Int. J. Tour. Res.* 23 1176–1190. 10.1002/jtr.2477

[B18] Chesbrough H. (2020). To recover faster from Covid-19, open up: managerial implications from an open innovation perspective. *Ind. Mark. Manag.* 88 410–413. 10.1016/j.indmarman.2020.04.010

[B19] Christodoulides G. Chernatony L. (2010). Consumer-based brand equity conceptualization and measurement: a literature review. *Int. J. Mark. Res.* 52 391–403.

[B20] Craney T. A. Surles J. G. (2007). Model-dependent variance inflation factor cutoff values. *Qual. Eng.* 14 391–403. 10.1081/QEN-120001878

[B21] Cruz-Milán O. (2021). Assessing the role of venturesomeness in a destination consumer-based brand equity model. *J. Hosp. Tour. Insights [Online ahead of print]* 10.1108/JHTI-09-2021-0264

[B22] Dimofte C. Goodstein R. Brumbaugh A. (2014). A social identity perspective on aspirational advertising: implicit threats to Self-Esteem and strategies to overcome them. *J. Consum. Psychol.* 25 416–430. 10.1016/j.jcps.2014.12.001

[B23] Erkmen E. (2018). Managing your brand for employees: understanding the role of organizational processes in cultivating employee brand equity. *Adm. Sci.* 8:52. 10.3390/admsci8030052

[B24] Fernández-Ruano M. L. Frías-Jamilena D. M. Polo-Peña A. I. Peco-Torres F. (2022). The use of gamification in environmental interpretation and its effect on customer-based destination brand equity: the moderating role of psychological distance. *J. Destin. Mark. Manag.* 23:100677. 10.1016/j.jdmm.2021.100677

[B25] Firmansyah M. R. Sumarwan U. Ali M. M. (2021). Marketing mix, brand equity, and purchase decisions of packaged rice products. *J. Manaj. Agribisnis* 18:240.

[B26] Fornell C. Larcker D. F. (1981). Evaluating structural equation models with unobservable variables and measurement error. *J. Mark. Res.* 18:39. 10.2307/3151312

[B27] Franke G. Sarstedt M. (2019). Heuristics versus statistics in discriminant validity testing: a comparison of four procedures. *Internet Res.* 29 430–447. 10.1108/IntR-12-2017-0515

[B28] Gielens K. Ma Y. Namin A. Sethuraman R. Smith R. J. Bachtel R. C. (2021). The future of private labels: towards a smart private label strategy. *J. Retail.* 97 99–115. 10.1016/j.jretai.2020.10.007

[B29] Gill-Simmen L. MacInnis D. J. Eisingerich A. B. Whan Park C. (2018). Brand-self connections and brand prominence as drivers of employee brand attachment. *AMS Rev.* 8 128–146. 10.1007/s13162-018-0110-6

[B30] Glaveli N. (2021). Two countries, two stories of CSR, customer trust and advocacy attitudes and behaviors? A study in the Greek and Bulgarian telecommunication sectors. *Eur. Manag. Rev.* 18 151–166. 10.1111/emre.12417

[B31] Górska-Warsewicz H. Żakowska-Biemans S. Stangierska D. Śwątkowska M. Bobola A. Szlachciuk J. (2021). Factors limiting the development of the organic food sector—perspective of processors, distributors, and retailers. *Agriculture* 11:882. 10.3390/agriculture11090882

[B32] Gounaris S. P. (2006). Internal-market orientation and its measurement. *J. Bus. Res.* 59 432–448. 10.1016/j.jbusres.2005.10.003

[B33] Hanaysha J. R. Al-Shaikh M. E. (2021). An examination of customer relationship management dimensions and employee-based brand equity: a study on ride-hailing industry in Saudi Arabia. *Res. Transp. Bus. Manag.* 100719. 10.1016/j.rtbm.2021.100719

[B34] Henseler J. Ringle C. M. Sarstedt M. (2015). A new criterion for assessing discriminant validity in variance-based structural equation modeling. *J. Acad. Mark. Sci.* 43 115–135. 10.1007/s11747-014-0403-8

[B35] Holiday S. Hayes J. L. Britt B. C. Lyu Y. (2021). The cause effect: the impact of corporate social responsibility advertising on cause consumer engagement behavior after brand affiliation ceases. *Int. J. Advert.* 40 199–224. 10.1080/02650487.2020.1769408

[B36] Hu Y. Xu A. Hong Y. Gal D. Sinha V. Akkiraju R. (2019). Generating business intelligence through social media analytics: measuring brand personality with consumer-, employee-, and firm-generated content. *J. Manag. Inf. Syst.* 36 893–930. 10.1080/07421222.2019.1628908

[B37] Islam T. Islam R. Pitafi A. H. Xiaobei L. Rehmani M. Irfan M. (2021). The impact of corporate social responsibility on customer loyalty: the mediating role of corporate reputation, customer satisfaction, and trust. *Sustain. Prod. Consum.* 25 123–135. 10.1016/j.spc.2020.07.019

[B38] Jalalzadeh S. R. Kazemi A. Ansari A. (2021). Developing a brand performance model based on customer-based brand equity in the market of Iran’s banking services. *Int. J. Bus. Excell.* 23 559–585.

[B39] Jamshidi D. Rousta A. (2021). Brand commitment role in the relationship between brand loyalty and brand satisfaction: phone industry in Malaysia. *J. Promot. Manag.* 27 151–176. 10.1080/10496491.2020.1809596

[B40] Jeon H. M. Yoo S. R. (2021). The relationship between brand experience and consumer-based brand equity in grocerants. *Serv. Bus.* 15 369–389. 10.1007/s11628-021-00439-8

[B41] Jogezai N. A. Baloch F. A. Jaffar M. Shah T. Khilji G. K. Bashir S. (2021). Teachers’ attitudes towards social media (SM) use in online learning amid the COVID-19 pandemic: the effects of SM use by teachers and religious scholars during physical distancing. *Heliyon* 7:e06781. 10.1016/j.heliyon.2021.e06781 33948511 PMC8080042

[B42] Kapoor S. Banerjee S. (2021). On the relationship between brand scandal and consumer attitudes: a literature review and research agenda. *Int. J. Consum. Stud.* 45 1047–1078. 10.1111/ijcs.12633

[B43] Keller K. L. (1993). Conceptualizing, measuring, and managing customer-based brand equity. *J. Mark.* 57 1–22. 10.2307/1252054

[B44] Kim E. J. Baloglu S. Henthorne T. L. (2021). Signaling effects of branded amenities on customer-based brand equity. *J. Hosp. Mark. Manag.* 30 508–527. 10.1080/19368623.2021.1846651

[B45] Kim J. Min J. E. Le L. H. (2021). Impacts of brand familiarity and brand responses on perceived brand credibility, similarity, and blog recommendation intention: a study of corporate blogs. *J. Fash. Mark. Manag. [Online ahead of print]* 10.1108/JFMM-09-2020-0189

[B46] King C. Grace D. (2005). Exploring the role of employees in the delivery of the brand: a case study approach. *Qual. Mark. Res. An Int. J.* 8 277–295. 10.1108/13522750510603343

[B47] King C. Grace D. (2008). Internal branding: exploring the employee’s perspective. *J. Brand Manag.* 15 358–372. 10.1057/palgrave.bm.2550136

[B48] King C. Grace D. (2009). Employee based brand equity: a third perspective. *Serv. Mark. Q.* 30 122–147. 10.1080/15332960802619082

[B49] King C. Grace D. (2010). Building and measuring employee-based brand equity. *Eur. J. Mark.* 44 938–971. 10.1108/03090561011047472

[B50] King C. Grace D. Funk D. (2013). Employee brand equity: scale development and validation. *J. Brand Manag.* 19 350–354. 10.1057/bm.2012.60

[B51] King C. Grace D. Funk D. C. (2012). Employee brand equity: scale development and validation. *J. Brand Manag.* 19 268–288. 10.1108/09526861211221518 22755484

[B52] Kotsi F. Pike S. Gottlieb U. (2018). Consumer-based brand equity (CBBE) in the context of an international stopover destination: perceptions of Dubai in France and Australia. *Tour. Manag.* 69 297–306.

[B53] Krizanova A. Lǎzǎroiu G. Gajanova L. Kliestikova J. Nadanyiova M. Moravcikova D. (2019). The effectiveness of marketing communication and importance of its evaluation in an online environment. *Sustainability* 11:7016.

[B54] Kumagai K. Nagasawa S. (2021). Moderating effect of brand commitment on apparel brand prestige in upward comparisons. *J. Glob. Fash. Mark.* 12 195–213. 10.1080/20932685.2021.1912630

[B55] Kumar V. Kaushal V. (2021). Perceived brand authenticity and social exclusion as drivers of psychological brand ownership. *J. Retail. Consum. Serv.* 61:102579. 10.1016/j.jretconser.2021.102579

[B56] Lee Y.-H. Hsiao C. Chan H.-Y. Lee I.-C. (2019). Explorations of employee-based brand equity in the banking industry from a perceived-leadership perspective. *Int. J. Bank Mark. [Online ahead of print]* 10.1108/IJBM-05-2019-0166

[B57] Li F. Larimo J. Leonidou L. C. (2021). Social media marketing strategy: definition, conceptualization, taxonomy, validation, and future agenda. *J. Acad. Mark. Sci.* 49 51–70. 10.1007/s11747-020-00733-3

[B58] Li S. Qu H. Wei M. (2021). Antecedents and consequences of hotel customers’ psychological ownership. *Int. J. Hosp. Manag.* 93:102773.

[B59] Liu A. X. Hsu C. H. C. Fan D. X. F. (2020). From brand identity to brand equity: a multilevel analysis of the organization–employee bidirectional effects in upscale hotels. *Int. J. Contemp. Hosp. Manag.* 32 2285–2304. 10.1108/IJCHM-08-2019-0680

[B60] Maleki Minbashrazgah M. Bagheri Garbollagh H. Varmaghani M. (2021). Brand-specific transactional leadership: the effects of brand-building behaviors on employee-based brand equity in the insurance industry. *Kybernetes [Online ahead of print]* 10.1108/K-03-2021-0201

[B61] Mangold W. G. Miles S. J. (2007). The employee brand: is yours an all-star? *Bus. Horiz.* 50 423–433. 10.1016/j.bushor.2007.06.001

[B62] Mckinley C. Mastro D. Warber K. (2014). Social identity theory as a framework for understanding the effects of exposure to positive media images of self and other on intergroup outcomes. *Int. J. Commun.* 8 1049–1068.

[B63] Mehta A. M. Tariq M. (2020). How brand image and perceived service quality affect customer loyalty through customer satisfaction. *Acad. Mark. Stud. J.* 24 1–10.

[B64] Mo Z. Liu M. T. Wong I. A. (2021). More than lip service to internal market orientation: a multilevel investigation of the role of internal service quality. *Int. J. Contemp. Hosp. Manag.* 33 2559–2585. 10.1108/IJCHM-10-2020-1133

[B65] Mody M. Hanks L. (2019). Consumption authenticity in the accommodations industry: the keys to brand love and brand loyalty for hotels and Airbnb. *J. Travel Res.* 59 173–189. 10.1177/0047287519826233

[B66] Niedermeier A. Emberger-Klein A. Menrad K. (2021). Drivers and barriers for purchasing green fast-moving consumer goods: a study of consumer preferences of glue sticks in Germany. *J. Clean. Prod.* 284:124804. 10.1016/j.jclepro.2020.124804

[B67] Nogueira M. Santarém F. Gomes S. (2020). Volunteer brand equity? Exploring the Adoption of Employee Brand Equity (EBE) dimensions to understand volunteers’ contributions to build nonprofit organizations’ brands. *J. Nonprofit Public Sect. Mark.* 32 73–104. 10.1080/10495142.2019.1689222

[B68] Olanipekun A. O. Omotayo T. Saka N. (2021). Review of the use of Corporate Social Responsibility (CSR) tools. *Sustain. Prod. Consum.* 27 425–435. 10.1016/j.spc.2020.11.012

[B69] Peñalba-Aguirrezabalaga C. Sáenz J. Ritala P. Vanhala M. (2021). Putting knowledge to work: the combined role of marketing and sales employees’ knowledge and motivation to produce superior customer experiences. *J. Knowl. Manag.* 25 2484–2505. 10.1108/JKM-09-2020-0727

[B70] Pillay P. Sibiya M. (2021). Evaluating the impact of brand experiences on customer-based brand equity for tournament title sponsors. *Afr. J. Mark. Manag.* 13 25–38.

[B71] Pina R. Dias A. (2021). The influence of brand experiences on consumer-based brand equity. *J. Brand Manag.* 28 99–115. 10.1057/s41262-020-00215-5

[B72] Poulis A. Wisker Z. (2016). Modeling employee-based brand equity (EBBE) and perceived environmental uncertainty (PEU) on a firm’s performance. *J. Prod. Brand Manag.* 25 490–503. 10.1108/JPBM-04-2015-0852

[B73] Pradhan S. Srivastava A. Mishra D. K. (2020). Abusive supervision and knowledge hiding: the mediating role of psychological contract violation and supervisor directed aggression. *J. Knowl. Manag.* 24 216–234. 10.1108/JKM-05-2019-0248

[B74] Prados-Peña M. B. Del Barrio-García S. (2021). Key antecedents of brand equity in heritage brand extensions: the moderating role of tourist heritage experience. *Eur. Res. Manag. Bus. Econ.* 27:100153. 10.1016/j.iedeen.2021.100153

[B75] Rajaobelina L. Prom Tep S. Arcand M. Ricard L. (2021). The relationship of brand attachment and mobile banking service quality with positive word-of-mouth. *J. Prod. Brand Manag.* 30 1162–1175. 10.1108/JPBM-02-2020-2747

[B76] Ramli N. A. Latan H. Nartea G. V. (2018). Why should PLS-SEM be used rather than regression? Evidence from the capital structure perspective. *Int. Ser. Oper. Res. Manag. Sci.* 267 171–209. 10.1007/978-3-319-71691-6_6

[B77] Rifi A. Mostafa R. B. (2021). Brand credibility and customer-based brand equity: a service recovery perspective. *J. Financ. Serv. Mark.* 27, 1–16. 10.1057/s41264-021-00097-x

[B78] Rovanto I. K. Bask A. (2021). Systemic circular business model application at the company, supply chain and society levels—A view into circular economy native and adopter companies. *Bus. Strateg. Environ.* 30 1153–1173. 10.1002/bse.2677

[B79] Schmidt H. J. Baumgarth C. (2018). Strengthening internal brand equity with brand ambassador programs: development and testing of a success factor model. *J. Brand Manag.* 25 250–265. 10.1057/s41262-018-0101-9

[B80] Serafini G. Parmigiani B. Amerio A. Aguglia A. Sher L. Amore M. (2020). The psychological impact of COVID-19 on the mental health in the general population. *QJM An Int. J. Med.* 113 229–235. 10.1093/qjmed/hcaa201 32569360 PMC7337855

[B81] Shah Alam S. Mohamed Sayuti N. (2011). Applying the theory of planned behavior (TPB) in halal food purchasing. *Int. J. Commer. Manag.* 21 8–20. 10.1108/10569211111111676

[B82] Sharma R. Yetton P. Crawford J. (2009). Estimating the effect of common method variance: the method—method pair technique with an illustration from TAM research. *Append. MIS Q.* 33 473–490. 10.2307/20650305

[B83] Siqueira J. R. Peña-García N. ter Horst E. Molina G. Villamil M. (2021). The role of brand commitment in the retail sector: the relation with open innovation. *J. Open Innov. Technol. Mark. Complex.* 7:154. 10.3390/joitmc7020154

[B84] Smith D. Jacobson J. Rudkowski J. L. (2021). Employees as influencers: measuring employee brand equity in a social media age. *J. Prod. Brand Manag.* 30 834–853. 10.1108/JPBM-03-2020-2821

[B85] Sonmez Cakir F. Adiguzel Z. (2022). Effects of innovative finance, strategy, organization and performance: a case study of company. *Int. J. Innov. Sci. [Online ahead of print]* 10.1108/IJIS-08-2021-0146

[B86] Sürücü Ö Öztürk Y. Okumus F. Bilgihan A. (2019). Brand awareness, image, physical quality and employee behavior as building blocks of customer-based brand equity: consequences in the hotel context. *J. Hosp. Tour. Manag.* 40 114–124. 10.1016/j.jhtm.2019.07.002

[B87] Tajfel H. Turner J. C. (2004). The social identity theory of intergroup behavior. *Polit. Psychol. Key Readings.* 276–293. 10.4324/9780203505984-16

[B88] Törmälä M. Saraniemi S. (2018). The roles of business partners in corporate brand image co-creation. *J. Prod. Brand Manag.* 27 29–40. 10.1108/JPBM-01-2016-1089

[B89] Troiville J. Hair J. F. Cliquet G. (2019). Definition, conceptualization and measurement of consumer-based retailer brand equity. *J. Retail. Consum. Serv.* 50 73–84. 10.1016/j.jretconser.2019.04.022

[B90] Wilson M. Robson K. Pitt L. (2021). Consumer subversion and its relationship to anti-consumption, deviant and dysfunctional behaviors, and consumer revenge. *Psychol. Mark.* 39 598–611. 10.1002/mar.21583

[B91] Xialong T. Gull N. Iqbal S. Asghar M. Nawaz A. Albashar G. (2021). Exploring & validating the effects of mega projects on infrastructure development influencing sustainable environment & project management. *Front. Psychol.* 12:663199. 10.3389/fpsyg.2021.663199 33935923 PMC8085247

[B92] Ye G. Hudders L. De Jans S. De Veirman M. (2021). The value of influencer marketing for business: a bibliometric analysis and managerial implications. *J. Advert.* 50 160–178. 10.1080/00913367.2020.1857888

[B93] Yingfei Y. Mengze Z. Zeyu L. Ki-Hyung B. Avotra A. A. R. N. Nawaz A. (2021). Green logistics performance and infrastructure on service trade and environment-measuring firm’s performance and service quality. *J. King Saud Univ.* 34:101683.

[B94] Yoo B. Donthu N. (2001). Developing and validating a multidimensional consumer-based brand equity scale. *J. Bus. Res.* 52 1–14. 10.1016/S0148-2963(99)00098-3

[B95] Yoshida M. Gordon B. S. James J. D. (2021). Social capital and consumer happiness: toward an alternative explanation of consumer-brand identification. *J. Brand Manag.* 28 481–494. 10.1057/s41262-021-00240-y

[B96] Yunpeng S. Khan Y. A. (2021). Understanding the effect of online brand experience on customer satisfaction in China: a mediating role of brand familiarity. *Curr. Psychol.* 10.1007/s12144-021-01706-7

[B97] Zarei A. Farjoo H. Bagheri Garabollagh H. (2021). How Social Media Marketing Activities (SMMAs) and brand equity affect the customer’s response: does overall flow moderate it?. *J. Internet Commer.* 1–23. 10.1080/15332861.2021.1955461

[B98] Zhang M. The Cong P. Sanyal S. Suksatan W. Maneengam A. Murtaza N. (2022). Insights into rising environmental concern: prompt corporate social responsibility to mediate green marketing perspective. *Econ. Res. Istraživanja.* 1–17. 10.1080/1331677X.2021.2021966

[B99] Zhou Z. Zheng F. Lin J. Zhou N. (2021). The interplay among green brand knowledge, expected eudaimonic well-being and environmental consciousness on green brand purchase intention. *Corp. Soc. Responsib. Environ. Manag.* 28 630–639. 10.1002/csr.2075

[B100] Zollo L. Filieri R. Rialti R. Yoon S. (2020). Unpacking the relationship between social media marketing and brand equity: the mediating role of consumers’ benefits and experience. *J. Bus. Res.* 117 256–267. 10.1016/j.jbusres.2020.05.001

